# ﻿ *Azygosporus* gen. nov., a synapmorphic clade in the family Ancylistaceae

**DOI:** 10.3897/mycokeys.85.73405

**Published:** 2021-12-31

**Authors:** Yue Cai1, Yong Nie*, Heng Zhao, ZiMin Wang, ZhengYu Zhou, XiaoYong Liu, Bo Huang

**Affiliations:** 1 College of Biology, Food and Environment, Hefei University, Hefei, 230601, China; 2 Anhui Provincial Key Laboratory for Microbial Pest Control, Anhui Agricultural University, Hefei 230036, China; 3 School of Civil Engineering and Architecture, Anhui University of Technology, Ma’anshan 243002, China; 4 College of Life Sciences, Shandong Normal University, Jinan 250014, China; 5 Institute of Microbiology, School of Ecology and Nature Conservation, Beijing Forestry University, Beijing 100083, China

**Keywords:** Entomophthorales, resting spores, saprophytic fungi, taxonomy

## Abstract

The fungal genus *Conidiobolus* sensu lato was delimited into four genera based on morphology and phylogeny. However, the taxonomic placement of *C.parvus* has not been determined until now. Here, we show that *C.parvus* belongs to a distinct lineage based on mitochondrial (mtSSU) and nuclear (*TEF1* and nrLSU) phylogenetic analyses. Phylogenetic analyses further revealed a new species as sister to *C.parvus*. We identified a synapomorphy uniting these lineages (azygospore production) that was not observed in other allied genera of the family Ancylistaceae, and erected a new genus *Azygosporus***gen. nov.** for this monophyletic group, with a new combination, *A.parvus***comb. nov.** as the type species. Within *Azygosporus*, the novel species *A.macropapillatus***sp. nov.** was introduced from China based on morphological characteristics and molecular evidence, which is characterized by its prominent basal papilla, in comparison to other closely related species, measuring 7.5–10.0×5.0–10.0 µm. Our study resolved the phylogenetic placement of *C.parvus* and improved the taxonomic system of the Ancylistaceae family.

## ﻿Introduction

*Conidiobolus* is the largest genus within the family Ancylistaceae, and includes mainly saprotrophs occurring in soil and plant debris, but also parasites of insects and animals (Vilela et al. 2010; Gryganskyi et al. 2012). After decades of study on more than 35 American and Indian *Conidoioblus* taxa (Drechsler 1952, 1953, 1954, 1955a, b, 1957, 1960, 1962; Srinivasan and Thirumalachar 1961, 1962a, b, 1967, 1968b), a numerical taxonomy was proposed that included 27 distinct species (King 1976a, b, 1977). Subsequently, the genus *Conidiobolus* was divided into three subgenera according to secondary conidia types (Ben-Ze’ev and Kenneth 1982). However, these morphologies can be difficult to distinguish and possess limited phylogenetic information that has limited our understanding of the evolution of *Conidiobolus* (Humber 1989). Since the division of *Conidiobolus*, several singe- and multi-locus phylogenetic analyses of the genus have shown that the proposed groups are polyphyletic (Jensen et al. 1998; Gryganskyi et al. 2013; Nie et al. 2018). The latest taxonomic revision of *Conidiobolus*, based on morphology and four genetic loci, revealed four lineages, and four genera (*Capillidium*, *Conidiobolus* sensu stricto, *Microconidiobolus* and *Neoconidiobolus*) were established (Nie et al. 2020a).

In addition to the size of primary conidia and the type of secondary conidia, resting spores are another character with taxonomic importance for recognizing *Conidiobolus* species (Humber 1997). Until now, four styles of resting spores have been reported: villose spores in *C.coronatus* and *C.lunulus* (Nie et al. 2020a; Goffre et al. 2020), zygospores and chlamydosporus in most members (King 1977; Nie et al. 2020a), and azygospores found only in *C.parvus* (Drechsler 1962; King 1977). Consequently, the taxonomic status of *C.parvus* remained uncertain, as a monotypic lineage in the most recent phylogeny analysis (Nie et al. 2020a, b).

Previous phylogenetic analyses have shown that is it not only *Conidiobolusparvus* that has questionable taxonomic placement. Our recent research has indicated that *C.lampragues* and *C.nanodes* should be assigned into the genus *Neoconidiobolus* (Nie et al. 2021). In this article, we describe a new genus, *Azygosporus* gen. nov., and a new species, *A.macropapillatus* sp. nov., and compare them to other allied taxa. We construct a multilocus (nrLSU, mtSSU, and *TEF1*) phylogeny that supports morphological results and confirm the treatment of ex-type cultures of *C.parvus* as a new combination in *Azygosporus* gen. nov., named *A.parvus* (Drechsler) B. Huang & Y. Nie, comb. nov.

## ﻿Materials and methods

### ﻿Isolates and morphology

Plant debris was collected from Tiantangzhai National Forest Parks (31°17'48" N, 115°78'18") and Fangtang (30°30'57" N, 118°42'17" E), Anhui Province, China. Isolations were carried out using the canopy-plating approach (King 1976a). A Petri dish with potato dextrose agar (PDA; potato 200 g, dextrose 20 g, agar 20 g, H_2_O 1000 ml) was inverted over the plant debris and incubated at 21 °C. We surveyed the PDA canopy daily for entomophthoroid fungi, which were transferred to new PDA for purification when detected. Morphological characters of mycelia, primary conidiophores, primary and secondary conidia, and resting spores were described with the method of King (1976a). The length and width of 35 primary conidia, 35 conidiophores and 50 azygospores were measured using an Olympus BX50 research microscope, and then photographed by an Olympus DP25 microscope-camera. Meanwhile, we observed the morphology of secondary conidia grown on 2% agar plates (agar 20 g, H_2_O 1000 ml) under a light microscope (Olympus BX50, Japan). The living culture was deposited in the Research Center for Entomogenous Fungi of Anhui Agricultural University, Anhui Province, China (RCEF), and duplicated in the China General Microbiological Culture Collection Center, Beijing, China (CGMCC). The dried cultures were deposited in the Herbarium Mycologicum Academiae Sinicae, Beijing, China (HMAS).

### ﻿DNA extraction, PCR amplification and sequencing

Fungal mycelia were incubated on PDA for 7 d at 21 °C. Total genomic DNA was extracted from fresh fungal mycelia by using a CTAB method followed Watanabe et al. (2010). We targeted three genetic loci for phylogenetic analyses: the large subunit of the nuclear ribosomal RNA (nrLSU), the small ribosomal subunit of the mitochondria (mtSSU), and translation elongation factor 1-alpha gene 1 (*TEF1*) were used for phylogenetic analysis. Details of the PCR primers and reactions can be found in Nie et al. (2020b). PCR products were purified according to the manufacturer protocol of Bioteke’s Purification Kit (Bioteke Corporation, Beijing, China). The sequences of the PCR products were determined on both strands by using dideoxy-nucleotide chain termination on an ABI 3700 automated sequencer at Shanghai Genecore Biotechnologies Company (Shanghai, China). Sequence chromatograms were proofread and assembled with Geneious 9.0.2 (http://www.geneious.com) and the nine new nucleotide sequences were deposited in GenBank (Table 1).

**Table 1.** Accession information for samples used in phylogenetic analyses.

Species	Strains*	GenBank accession numbers		
		nucLSU	*TEF1*	mtSSU
*Azygosporusmacropapillatus*	RCEF 4444	MZ542004	MZ555648	MZ542277
*A.macropapillatus*	RCEF 6334	MZ542005	MZ555649	MZ542278
*A.macropapillatus*	CGMCC 3.16068 (T)	MZ542006	MZ555650	MZ542279
*A.parvus*	ATCC 14634 (T)	KX752051	KY402207	MK301192
*Capillidiumadiaeretum*	CGMCC 3.15888	MN061284	MN061481	MN061287
*Ca.bangalorense*	ARSEF 449 (T)	DQ364204	–	DQ364225
*Ca.heterosporum*	RCEF 4430	JF816225	JF816239	MK301183
*Ca.lobatum*	ATCC 18153 (T)	JF816218	JF816233	MK301187
*Ca.rhysosporum*	ATCC 12588 (T)	JN131540	JN131546	MK301195
*Conidiobolusbifurcatus*	CGMCC 3.15889 (T)	MN061285	MN061482	MN061288
*C.brefeldianus*	ARSEF 452 (T)	EF392382	–	EF392495
*C.chlamydosporus*	ATCC 12242 (T)	JF816212	JF816234	MK301178
*C.coronatus*	NRRL 28638	AY546691	DQ275337	–
*C.dabieshanensis*	CGMCC 3.15763 (T)	KY398125	KY402206	MK301180
*C.firmipilleus*	ARSEF 6384	JX242592	–	JX242632
*C.gonimodes*	ATCC 14445 (T)	JF816221	JF816226	MK301182
*C.humicolus*	ATCC 28849 (T)	JF816220	JF816231	MK301184
*C.iuxtagenitus*	ARSEF 6378 (T)	KC788410	–	–
*C.khandalensis*	ATCC 15162 (T)	KX686994	KY402204	MK301185
*C.lichenicolus*	ATCC 16200 (T)	JF816216	JF816232	MK301186
*C.marcosporus*	ATCC 16578 (T)	KY398124	KY402209	MK301188
*C.megalotocus*	ATCC 28854 (T)	MF616383	MF616385	MK301189
*C.mycophagus*	ATCC 16201 (T)	JX946694	JX946698	MK301190
*C.mycophilus*	ATCC 16199 (T)	KX686995	KY402205	MK301191
*C.polyspermus*	ATCC 14444 (T)	MF616382	MF616384	MK301193
*C.polytocus*	ATCC 12244 (T)	JF816213	JF816227	MK301194
*C.taihushanensis*	CGMCC 3.16016 (T)	MT250088	MT274290	MT250086
*C.variabilis*	CGMCC 3.16015 (T)	MT250087	MT274289	MT250085
*Eryniaconica*	ARSEF 1439	EF392396	–	EF392506
*Entomophthoramuscae*	ARSEF 3074	DQ273772	DQ275343	–
*Microconidiobolusnodosus*	ATCC 16577 (T)	JF816217	JF816235	MK333391
*M.paulus*	ARSEF 450 (T)	KC788409	–	–
*M.terrestris*	ATCC 16198 (T)	KX752050	KY402208	MK301199
*M.undulatus*	ATCC 12943 (T)	JX946693	JX946699	MK301201
*Neoconidioboluscouchii*	ATCC 18152 (T)	JN131538	JN131544	MK301179
*N.kunyushanensis*	CGMCC 3.15890 (T)	MN061286	MN061483	MN061289
*N.lamprauges*	CBS 461.97	MH874268	–	–
*N.lachnodes*	ARSEF 700	KC788408	–	–
*N.mirabilis*	CGMCC 3.17763 (T)	MH282852	MH282853	MK333392
*N.nanodes*	CBS 154.56 (T)	MH869096	–	–
*N.osmodes*	ARSEF 79	EF392371	–	DQ364219
*N.pachyzygosporus*	CGMCC 3.17764 (T)	KP218521	KP218524	MK333393
*N.sinensis*	RCEF 4952 (T)	JF816224	JF816238	MK301196
*N.stilbeus*	RCEF 5584 (T)	KP218522	KP218525	MK301197
*N.stromoideus*	ATCC 15430 (T)	JF816219	JF816229	MK301198
*N.thromboides*	ATCC 12587 (T)	JF816214	JF816230	MK301200

*ARSEF, ARS Entomopathogenic Fungus Collection (Ithaca, U.S.A.). ATCC, American Type Culture Collection (Manassas, U.S.A). CBS, Westerdijk Fungal Biodiversity Institute (Utrecht, The Netherlands). CGMCC, China General Microbiological Culture Collection Center (Beijing, China). FSU, Jena Microbial Resource Collection (Friedrich-Schiller-University of Jena, Germany). NRRL, ARS Culture Collection (Peoria, U.S.A). RCEF, Research Center for Entomogenous Fungi (Hefei, China). T = ex-type.

### ﻿Phylogenetic analyses

We downloaded nrLSU, mtSSU, and *TEF1* sequences of 5 *Capillidium* species, 19 *Conidiobolus* s.s. strains, four *Microconidiobolus* strains, 12 *Neoconidiobolus* species, *C.parvus*, and two outgroup taxa (*Entomophthoramuscae* and *Eryniaconica*) from GenBank. Individual sequences of each locus were aligned using MUSCLE 3.8.31 (Edgar 2004) and concatenated matrices were assembled by SequenceMatrix 1.7.8 (Vaidya et al. 2011). We partitioned the concatenated matrix by selecting the best model of sequence evolution for each gene according to the Akaike Information Criterion (AIC) using Modeltest 3.7 (Posada and Crandall 1998). We then conducted a Maximum Likelihood (ML) phylogenetic analysis using the best model using RAxML 8.1.17 with 1000 bootstrap replicates (Stamatakis 2014). We also built a phylogeny using Bayesian Inference (BI) with MrBayes 3.2.2 (Ronquist and Huelsenbeck 2003). We ran four Markov chains for 400,000 generations, sampling every 100^th^ generation, and chains were run until the standard deviation of split frequencies fell below 0.01. Maximum Parsimony (MP) analyses were performed with PAUP* 4.0b10 (Swofford 2002) using the heuristic research option with random stepwise addition, random taxon addition of sequences, tree bisection and reconnection (TBR) as the branch swapping algorithm, and 1000 replicates. All characters were weighted equally and character state transitions were treated as unordered. Parameters measured for parsimony included tree length (TL), consistency index (CI), rescaled consistency index (RC), retention index (RI), and homoplasy index (HI). The sequence matrix was deposited at TreeBase (No. S28467). Phylogenetic trees were viewed in TreeView (Page 1996) and edited in FigTree 1.4 (Rambaut 2012).

## ﻿Results

### ﻿Phylogenetic analyses

**Figure 1.**
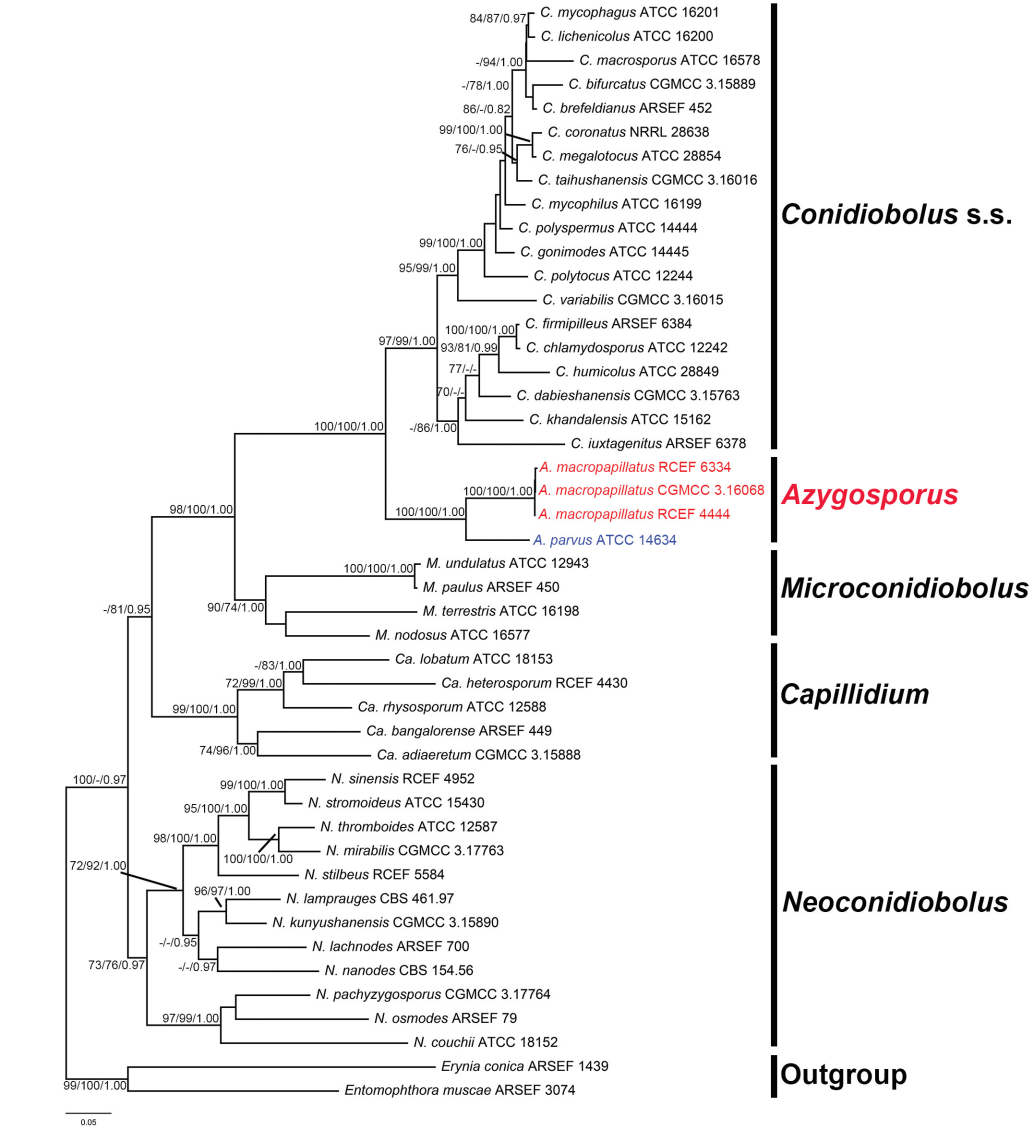
ML tree of *Coniobolus* s.l. using nrLSU + *TEF1* + mtSSU sequences. *Entomophthoramuscae* and *Eryniaconica* are selected as outgroups. Support for each node is shown as MP bootstrap support/ML bootstrap support/Bayesian posterior probability (MPBS/MLBS/BPP) for nodes with MPBS≧70%, MLBS≧70%) and BPP≧0.95. The new genus, *Azyosporus*, and new species, *A.macropapillatus*, are shown in red, and the new combination is shown in blue.

The total alignment length of the 46 taxa was 2,002: nrLSU, 1–1,095; *TEF1*, 1,096–1,597; and mtSSU, 1,598–2,002. The concatenated matrix contained 957 parsimony-informative and 225 parsimony-uninformative sites. The MP tree had a length of 5,463 with CI = 0.3815, RC = 0.2467, RI = 0.6404, and HI = 0.6471. We found that the optimal model of sequence evolution for nrLSU and *TEF1* were GTR+I+G4, while TVM+I+G4 was selected for mtSSU, and the resulting BI, ML, and MP trees had similar topologies; the ML tree was selected to represent the phylogeny with MP/ML/BI support values (Fig. 1). Samples from *Azygosporusmacropapillatus* sp. nov. were sister to *C.parvus* (= *A.parvus*) in a single clade mostly related to the genus *Conidiobolus* s.s. in the phylogenetic tree. Both the clades of *Azygosporus* gen. nov. and *A.macropapillatus* sp. nov. were monophyletic with strong support (100/100/1.00).

### ﻿Taxonomy

#### ﻿ Azygosporus

Taxon classificationFungiEntomophthoralesAncylistaceae

B. Huang & Y. Nie gen. nov.

3C5FFB86-334D-5721-A5A0-AB685C48A3C4

MycoBank No: 840849

##### Etymology.

Referring to produce azygospores.

##### Type species.

*Azygosporusparvus* (Drechsler) B. Huang & Y. Nie.

##### Description.

Mycelia colorless. Primary conidiophores simple, bearing single primary conidia. Primary conidia forcibly discharged multinucleate, colourless, globose to subglobose, small, less than 22.5 μm. Producing only globose or subglobose replicative conidia, similar to and smaller than primary conidia. Azygospores formed in the middle region of the old hyphal segments. Mature azygospores colourless or yellowish, smooth, without thickening or less thickening (0.5–1.2 μm).

##### Notes.

*Azygosporus* is strongly supported as monophyletic and is distinguished from other Ancylistaceae lineages by the synapomorphy of azygospore production. Therefore, we classify this lineage as a new genus, named *Azygosporus* gen. nov. *Azygosporus* currently contains only two members: *C.parvus* (= *A.parvus*) and *A.macropapillatus* sp. nov. (Fig. 1). Morphologically, *Azygosporus* is most similar to *Microconidiobolus*, which forms small primary conidia (less than 22.5 μm) (Table 2). However, the synapomorphy of azygospore production clearly distinguishes *Azygosporus* from *Microconidiobolus* and other allied genera of the family.

**Table 2.** Morphological measurements of *A.macropapillatus* and other related species.

Species	Growth rate (mm/d) at 21oC on PDA	Diameter of mycelia (μm)	Primary conidiophores (μm)	Primary conidia (μm)	Basal papilla (μm)	Resting spores (μm)	References
*A.macropapillatus*	5.7–7.7	3.0–7.5	37.0–150.0×5.0–8.5	16.5–22.5×12.0–19.0	7.5–10.0×5.0–10.0	azygosporus, 25.0–30.0×27.0–34.0	This article
*A.parvus*	1.5	1.4–8.0 (3.5–5)	15.0–30.0×3.0–8.0	6.0–20.0×4.5–17.0	1.5–6.0×1.5–4.5	azygosporus, 20.0–25.0×8.0–20.0	Drechsler 1962
*M.nodosus*	7.1	3.5–6.5	30.0–50.0	17.0–22.0×13.0–16.0	2.5–5.0×1.5–2.5	chlamydosporus	Srinivasan and Thirumalachar 1967; King 1977
*M.paulus*	1.3–3.3	1.5–7.0 (4.0–5.0)	15.0–30.0×3.5–7.0	5.0–19.0×4.0–14.0	2.0–7.0×1.0–5.0	zygosporus, 10.0–15.0	Drechsler 1957
*M.terrestris*	2.6	2.8–4.5	15.0–80.0 ×3.0–5.0	8.0–12.0	2.0–4.0×1.5–2.0	chlamydosporus	Srinivasan and Thirumalachar 1968a; King 1977
*N.lamprauges*	less than 5.0	3.0–8.0 (4.0–7.0)	25.0–100.0 (25.0–50.0)×4.0–8.0 (5.0–15.0)	15.0–22.0×12.5–20.0	2.5–7.0×1.5–4.0	zygosporus, 12.0–18.0	Drechsler 1953
*N.kunyushanensis*	8.3–10.0	3.5–9.0	62.0–121.0×7.0–12.0	15.0–21.0×13.0–17.0	4.0–8.0×1.0–4.0	zygosporus, 12.0–25.0	Nie et al. 2021
*N.pachyzygosporus*	12.0	3.0–14.0	34.0–156.0×6.0–12.0	15.5–23.0×11.0–18.0	3.0–5.0×1.0–4.0	zygosporus, 15.0–25.0	Nie et al. 2018

#### ﻿ Azygosporus parvus

Taxon classificationFungiEntomophthoralesAncylistaceae

(Drechsler) B. Huang & Y. Nie comb. nov.

3C3F8153-5748-5C5A-BAA7-69F154F09E09

MycoBank No: 840850

Conidiobolus parvus Drechsler, Bull. Torrey bot. Club 89: 233 (1962) Basionym.

##### Description.

Refer to Drechsler (1962).

##### Host and distribution.

Isolated from decaying leaves in Maryland, United States.

##### Notes.

The ex-type living culture is ATCC 14634 (United States, Maryland, Cumberland, 4 November 1962, Drechsler). It was reported to produce azygospores in *Conidiobolus* (King 1977); therefore, we recognize it as the type species of the genus *Azygosporus* gen. nov.

#### ﻿ Azygosporus macropapillatus

Taxon classificationFungiEntomophthoralesAncylistaceae

B. Huang & Y. Nie sp. nov.

7B9EC33A-B4EA-53E4-BC06-FE4913B082C7

MycoBank No: 840548

Fig. 2

##### Etymology.

*macropapillatus* (Lat.), named by its prominent basal papilla.

##### Host and known distribution.

Isolated from plant debris and mosses in Anhui Province, China.

##### Type specimens examined.

China, Anhui Province, Ningguo City, Fangtang Town, 30°30'57" N, 118°42'17" E, from plant debris, 12 Nov 2020, *Y. Nie*, HMAS 350621, holotype, culture ex-holotype *CGMCC 3.16068* (= *RCEF 6680*). GenBank: nrLSU = MZ542006; *TEF1* = MZ555650; mtSSU = MZ542279.

**Figure 2.**
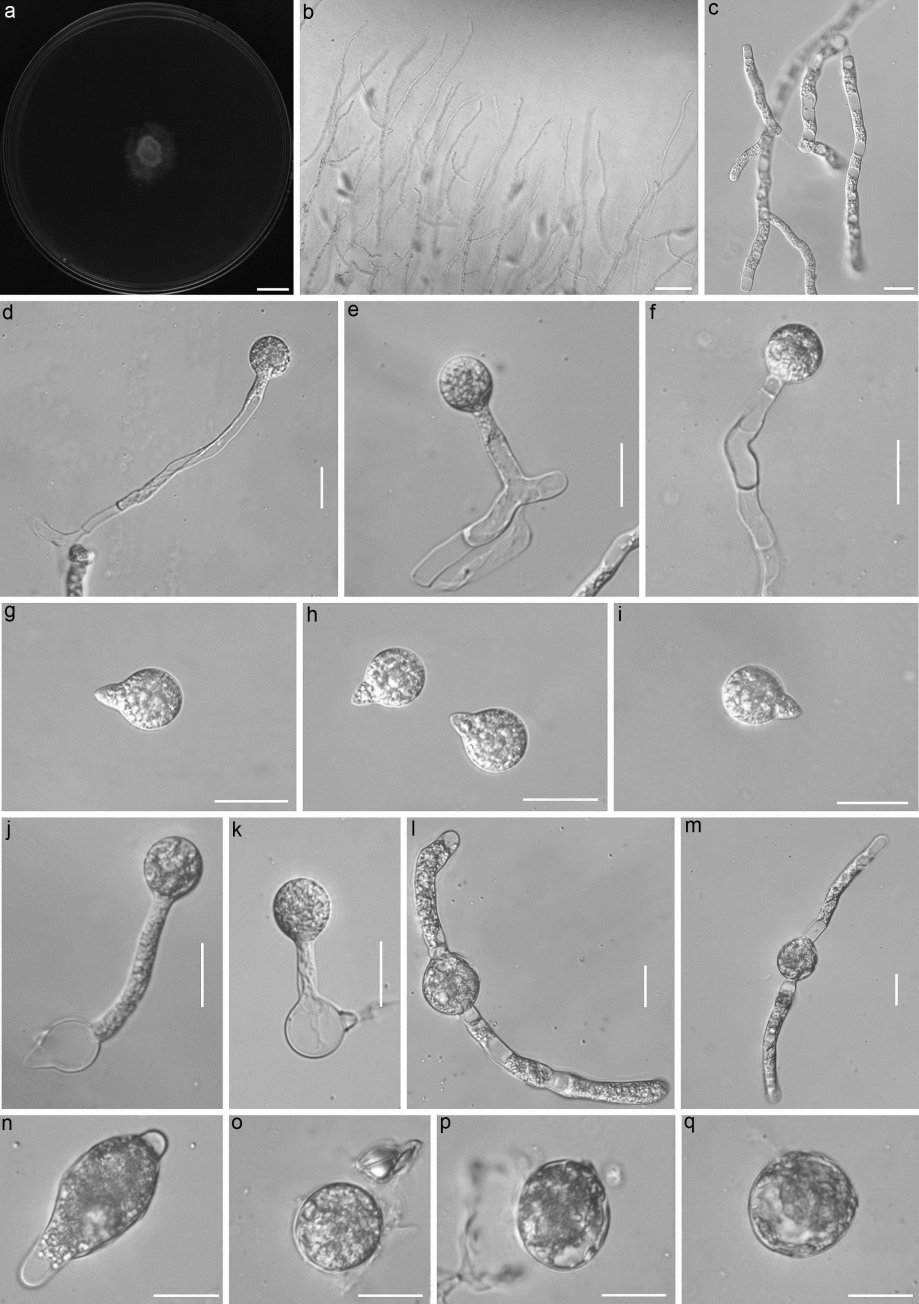
Morphological characters of *Azygosporusmacropapillatus*: a) colony on PDA after 3 d at 21 °C, b) mycelia rarely branched at the colony edge, c-f) primary conidiophores bearing primary conidia, g-h) Primary conidia with prominent basal papillum, j-k) secondary conidia arising from primary conidia, i-m) azygospores formed in the middle region of the old hyphal segment, n) immature azygospore, and o-q) mature azygospores. Scale bars: a) 10 mm, b) 100 μm, and c-q) 20 μm.

##### Additional specimens examined.

China, Anhui Province, Jinzhai County, Tiantangzhai National Forest Park, 31°20'68" N, 115°81'25" E, from mosses, 6 Nov 2008, *C.F. Wang*, culture *RCEF 4444*. GenBank: nrLSU = MZ542004; *TEF1* = MZ555648; mtSSU = MZ542277. China, Anhui Province, Jinzhai County, Tiantangzhai National Forest Park, 31°17'34" N, 115°78'13" E, from plant debris, 3 Dec 2015, *Y. Nie* and *X.X. Tang*, culture *RCEF 6334*. GenBank: nrLSU = MZ542005; *TEF1* = MZ555649; mtSSU = MZ542278.

##### Description.

Colonies white, reaching ca 17.0–23.0 mm diameter on PDA after 3 d at 21°C. Mycelia colorless, 3.0–7.5 µm wide, usually unbranched at the colony edge. Primary conidiophores colorless, without widening upward near the tip, unbranched and producing a single conidium, 37.0–150.0 × 5.0–8.5 µm. Primary conidia forcibly discharged, colorless, subglobose, 12.0–19.0 µm wide and 16.5–22.5 µm long, most primary conidia possessed a prominent basal papilla 5.0–10.0 µm wide and 7.5–10.0 µm long. Secondary conidia arising from the primary ones with a similar shape and a smaller size. Resting spores (azygospores) observed after 10 d, and the young spores formed in the middle region of the old hyphal segments. The young spores enlarge gradually to form mature azygpspores with less thickening. Mature azygospores colorless, subglobose 25.0–30.0 × 27.0–34.0 μm with a wall 0.5–1.0 μm thick.

##### Notes.

Morphologically, *Azygosporusmacropapillatus* sp. nov. has conidial dimensions similar to six *Conidiobolus* s.l. species without capilliconidia and microconidia: *C.parvus*, *M.nodosus*, *M.paulus*, *N.kunyushanensis*, *N.lamprauges*, and *N.pachyzygosporus* (Drechsler 1953, 1957, 1962; Srinivasan and Thirumalachar 1967; Nie et al. 2018, 2021). However, *A.macropapillatus* sp. nov. produces a prominent basal papilla of primary conidia that differs from other related species (see detailed morphological comparisons in Table 2). *A.macropapillatus* sp. nov. forms azygospores most closely resembling those of *C.parvus* (= *A.parvus*), which is its closest known relative with robust support (100/100/1.00). *A.macropapillatus* sp. nov. is distinguished from *C.parvus* (= *A.parvus*) by its longer primary conidiophore and its prominent basal papilla.

## ﻿Discussion

The genus *Microconidiobolus*, typified by *M.paulus*, was recently established as a monotypic genus based on its small discharged primary conidia (less than 20 μm) (Nie et al. 2020a). Besides the species shown in Table 2, we note that overlapping small primary conidial dimensions occur in other related genera such as, *Capillidiumpumilum* (7.3–14 × 9–18 µm) (Drechsler 1955a) and *Conidioboluskhandalensis* (15–18 × 17–21 µm) (Srinivasan and Thirumalachar 1962b). Therefore, taxonomic definitions in this fungal group, as in all life, should be revised to follow phylogenetic relationships. Phylogenetically, *Ca.pumilum* and *C.khandalensis* were distinct from *Microconidiobolus* spp. and *C.parvus* (= *A.parvus*) (Nie et al. 2018; 2020a). However, our previous phylogeny recovered *C.parvus* (= *A.parvus*) as distinct lineage within *Conidiobolus* s.s. (Nie et al. 2018; 2020b), but its taxonomic placement was uncertain due to its affinity with members of *Microconidiobolus*.

The phylogeny presented here (Fig. 1) is congruent with previous studies (Nie et al. 2018; 2020b) that investigated the placement of *C.parvus* (= *A.parvus*). Here, we demonstrate that *C.parvus* (= *A.parvus*) is sister to a new taxon, *A.macropapillatus* sp. nov., in a clade most closely related to *Conidiobolus* s.s., which we name *Azygosporus*. The primary character distinguishing *Azygosporus* from *Conidiobolus* s.s. is the production of microspores. Furthermore, *Azygosporus* can be distinguished from the related genus *Microconidiobolus*, by the production of azygospores, while members of *Microconidiobolus* form zygospores or chlamydospores. Azygospores were not observed in other related genera of the family Ancylistaceae. Consequently, we proposed a new genus, *Azygosporus* gen. nov., based on morphology and phylogeny. In addition, *C.parvus* (= *A.parvus*) was recognized as a new combination in the genus *Azygosporus* gen. nov. and introduced *A.macropapillatus* sp. nov. by its prominent basal papilla.

For decades, most published *Conidiobolus* species had been described by only one strain, with the exception of some pandemic species (e.g., *C.coronatus*, *N.osmodes*, and *N.thromboides*) (Nie et al. 2021). Unfortunately, the type species *C.utriculosus* Brefeld, along with other important ex-types are missing, which makes it difficult to determine the exact taxonomic placement of some questionable *Conidiobolus* spp. Expanding our descriptions of fungal diversity and improving the taxonomic system of this fungal group are continuing goals. Herein, we introduced a new genus and a new species, which are contributions to fungal taxonomy.

## Supplementary Material

XML Treatment for Azygosporus

XML Treatment for Azygosporus parvus

XML Treatment for Azygosporus macropapillatus
